# Concentration and size distribution data of silicon nitride nanoparticles measured using nanoparticle tracking analysis

**DOI:** 10.1016/j.dib.2017.09.011

**Published:** 2017-09-15

**Authors:** Saurabh Lal, Richard M. Hall, Joanne L. Tipper

**Affiliations:** University of Leeds, UK

## Abstract

This article refers to the paper “A novel method for isolation and recovery of ceramic nanoparticles and metal wear debris from serum lubricants at ultra-low wear rates” (Lal et al., 2016) [1] and describes the concentration and size distribution data of silicon nitride nanoparticles measured using nanoparticle tracking analysis (NTA). A NanoSight LM10 instrument was used to capture the video data of silicon nitride nanoparticles moving under Brownian motion in the water. The video data was then analyzed using the NanoSight NTA software. This article also describes a methodology for calculating the percentage recovery of a nanoparticle isolation process.

**Specifications Table**

**Table:** 

Subject area	*Nanoparticle Characterization*
More specific subject area	*Ceramic nanoparticle characterization and particle recovery*
Type of data	*Brownian motion video of silicon nitride nanoparticles in water captured by the NanoSight LM10 instrument. CSV file containing the concentration and size distribution data of silicon nitride nanoparticles dispersed in water.*
How data was acquired	*NanoSight LM10 (Malvern Instruments, UK) and NTA v 3.00 software*
Data format	*Raw*
Experimental factors	• *Silicon nitride nanoparticles were sonicated for 10 min to aid particle dispersion before taking NTA measurements.*
	• *The measurements were taken at room temperature.*
	• *The viscosity of water was assumed to be 1 cP.*
	• *Five measurements were taken for analysis of each sample.*
Experimental features	*Size distribution, concentration distribution and average concentration data of silicon nitride nanoparticles.*
Data source location	*University of Leeds, Leeds, UK*
Data accessibility	*Data are presented in this article*

**Value of the data**

• Video data showed that nanoparticle tracking analysis (NTA) can be used to visualize silicon nitride nanoparticles dispersed in water.
• Concentration and size distribution data demonstrates that silicon nitride nanoparticles dispersed in water can be characterized by NTA.
• NTA is suitable for measuring particle characteristics at low particle concentrations (10^7^–10^9^ particles per ml).
• The concentration of particles before and after a process can be used for estimating the percentage loss or percentage recovery of particles.

## 1 Data

The video file (Nanosight_video_Si3N4_NPs.mp4) is a video of silicon nitride (Si_3_N_4_) nanoparticles moving under Brownian motion in the water. The concentration-size distribution data of the Si_3_N_4_ nanoparticles is reported in Table 1.

**Table 1:** Concentration size distribution of silicon nitride nanoparticles dispersed in water.

*Particle Size (nm)*	*Concentration (E6 particles/ml)*
0–00	110.94
100–200	386.07
200–300	162.26
300–400	11.60
400–500	1.63
500–600	0.22
600–700	0.15
700–800	0.15
800–900	0.01
900–1000	0.00
Average total particle concentration	673.07

Mean Size = 162.37 nm	
Mode Size = 142 nm	

Supplementary material related to this article can be found online at doi: 10.1016/j.dib.2017.09.011.

The following is the Supplementary material related to this article Movie S1.

Movie S1 This video shows the Brownian motion of silicon nitride nanoparticles in water captured by the NanoSight LM10 instrument..

## 2 Experimental design, materials and methods

### 2.1 Preparation of the particles

Silicon nitride nanoparticles (<50 nm; Sigma, UK) were added to sterile water (Baxter, UK) to make 1 (mg ml^−1^) suspensions.

The suspensions were then diluted with sterile water to achieve an optimum concentration range of 10^7^–10^9^ particles per ml (approximately 20–100 particles in the field of view of the NanoSight video window). Nanoparticles were dispersed by sonication for 10 min in an ultrasonic bath (USC300T, VWR UK) before introducing them into the NanoSight flow cell.

### 2.2 Video capture and analysis

A minimum of five 30 s videos of the particles moving under Brownian motion were captured by NanoSight. The videos were then analyzed for size distribution and particle concentration using the built-in NTA v 3.0 software.

### 2.3 Measurement of particle concentration and percentage recovery

The concentration values obtained after analysis were averaged to obtain a mean concentration value, which was then multiplied by the dilution factor (described in Section 2.1) to obtain the initial concentration of silicon nitride particles dispersed in water.

When the size distribution of nanoparticles remains unaffected by a process, such as a particle isolation procedure described in Lal et al. [1], the percentage recovery of particles suspended in a liquid can be calculated using the following formula:

(1) $$\mathrm{Percentage}\;\mathrm{Recovery}=\frac{\mathrm{Average}\;\mathrm{Final}\;\mathrm{Concentration}(\mathrm{number}\;\mathrm{of}\;\mathrm{perticles}/\mathrm{ml})}{\mathrm{Average}\;\mathrm{Initial}\;\mathrm{Concentration}(\mathrm{number}\;\mathrm{of}\;\mathrm{particles}/\mathrm{ml})}\times 100$$
